# A Sensitive and Selective Colorimetric Method Based on the Acetylcholinesterase-like Activity of Zeolitic Imidazolate Framework-8 and Its Applications

**DOI:** 10.3390/molecules27217491

**Published:** 2022-11-03

**Authors:** Guo-Ying Chen, Zheng-Ming Qian, Shi-Jun Yin, Xi Zhou, Feng-Qing Yang

**Affiliations:** 1 School of Chemistry and Chemical Engineering, Chongqing University, Chongqing 401331, China; 2 College of Medical Imagine Laboratory and Rehabilitation, Xiangnan University, Chenzhou 423000, China; 3 Dongguan HEC Cordyceps R&D Co., Ltd., Dongguan 523850, China

**Keywords:** zeolitic imidazolate framework-8, acetylcholinesterase, copper ion, sulfathiazole, glucose

## Abstract

In this study, a simple colorimetric method was established to detect copper ion (Cu^2+^), sulfathiazole (ST), and glucose based on the acetylcholinesterase (AChE)-like activity of zeolitic imidazolate framework-8 (ZIF-8). The AChE-like activity of ZIF-8 can hydrolyze acetylthiocholine chloride (ATCh) to thiocholine (TCh), which will further react with 5,5′-dithiobis (2-nitrobenzoic acid) (DTNB) to generate 2-nitro-5-thiobenzoic acid (TNB) that has a maximum absorption peak at 405 nm. The effects of different reaction conditions (buffer pH, the volume of ZIF-8, reaction temperature and time, and ATCh concentration) were investigated. Under the optimized conditions, the value of the Michaelis-Menten constant (*K_m_*) is measured to be 0.83 mM, which shows a high affinity toward the substrate (ATCh). Meanwhile, the ZIF-8 has good storage stability, which can maintain more than 80.0% of its initial activity after 30 days of storage at room temperature, and the relative standard deviation (RSD) of batch-to-batch (*n* = 3) is 5.1%. The linear dependences are obtained based on the AChE-like activity of ZIF-8 for the detection of Cu^2+^, ST, and glucose in the ranges of 0.021–1.34 and 5.38–689.66 µM, 43.10–517.24 µM, and 0.0054–1.40 mM, respectively. The limit of detections (LODs) are calculated to be 20.00 nM, 9.25 µM, and 5.24 µM, respectively. Moreover, the sample spiked recoveries of Cu^2+^ in lake water, ST in milk, and glucose in strawberry samples were measured, and the results are in the range of 98.4–115.4% with the RSD (*n* = 3) lower than 3.3%. In addition, the method shows high selectivity in the real sample analysis.

## 1. Introduction

Nowadays, enzyme-mimicking catalytic nanomaterial (nanozyme) has been rapidly developed, attracting much attention in many fields. The nanozyme can make up for several shortcomings of biological enzymes, such as poor stability in harsh environments, difficulty in recycling, and high-cost of production. The frequently used nanozymes include metal oxides, metal sulfides, noble metals, and metal-organic frameworks (MOFs) [1,2,3]. MOFs, owning uniform porous structure, high surface area, porosity, and good chemical stability, are composed of organic ligands and metal nodes, which have been intensively used in the analysis of gases, ions, aromatic compounds, and bioactive species based on optical, electrochemical, mechanical or other sensing methods [4]. For example, He, et al. [5] successfully synthesized a novel 3D ruthenium-based metal-organic gels (Ru-MOGs) with fibrillar network structure using a facile one-step strategy at room temperature, which was used in the detection of glucose with a good linearity in the range of 0.02–5 μM, and the limit of detection (LOD) is 9 nM. Sun et al. [6] synthesized an Eu-MOF ([Eu_2_L_2_(DMF)-(H_2_O)_2_] 2DMF H_2_O) as a turn-off probe for the detection of ferric ion (Fe^3+^) and copper ion (Cu^2+^) in N, N-dimethylformamide (DMF), and the LODs are 12.2 µM and 18.3 µM, respectively. Nevertheless, the synthesis conditions of most MOF materials require high temperature and pressure, large amounts of organic reagents, and long synthesis time. Among the reported MOFs, zeolitic imidazolate framework-8 (ZIF-8) shows a relatively high porous, robust structure, and multiple enzyme activities. In addition, the synthesis process of ZIF-8 is green and simple.

Cu^2+^, an important cofactor or a structural component of many enzymes and other proteins in living organisms, plays an important role in several physiological processes, such as cellular respiration, hematopoietic function, bone formation, prevention of cardiovascular diseases, and connective tissue development [7]. Because of its widespread applications in agriculture and industry, Cu^2+^ continues to be one of the major components of environmental pollutants. Excessive Cu^2+^ induces damages to liver, brain, kidney and other tissues, and further result in serious neurological diseases, such as Wilson’s, Parkinson’s, and Alzheimer’s diseases [8]. The World Health Organization requires that the concentration of Cu^2+^ in drinking water should be less than 2.0 mg/L, and the safe concentration limit of Cu^2+^ in drinking water stipulated by the Chinese Ministry of Health is 1.0 mg/L [9]. Therefore, it is necessary to establish a fast, sensitive, and convenient method for the determination of Cu^2+^.

Sulfathiazole (ST), a kind of synthetic antimicrobial agent that contains the sulfonamide group, is active against a broad spectrum of gram-positive and gram-negative bacteria, which is a kind of antibacterial drug used in farm animals for the treatment of various bacterial infections. Prolonged or irregular using, or prolonged contacting to the residues of ST may lead to antibiotic resistance in both veterinary and human applications [10]. Moreover, ST is found to be carcinogenic and has relatively long half-life. The widespread use of antibiotics will not only affect the ecological system but also risk the contamination of food, and their metabolites endanger the health of the consumers [11]. Therefore, it is necessary to monitor ST in real-time.

Glucose is the energy source and metabolic intermediate product of living cells, which is closely related to the metabolism of the endocrine system. Glucose is one of the most important monosaccharides in almost all kinds of fruits, which is used as an indicator of fruit quality [12]. Consuming fruits containing high sugar will increase the blood sugar level and blood sugar load of patients with diabetes [13]. The detection of glucose concentration is vital in many fields, such as medical diagnosis and the food industry [14]. Thus, the development of a sensitive glucose sensor is essential for practical applications.

**Figure 1:**
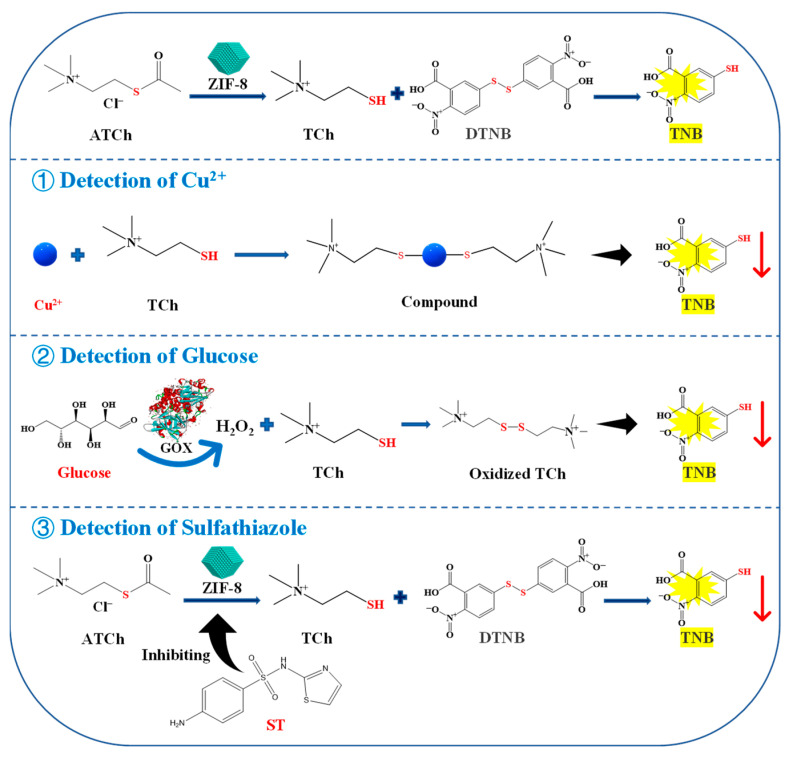
Schematic illustration for the detection of Cu^2+^, glucose, and ST based on the AChE-like activity of ZIF-8. ATCh, acetylthiocholine chloride; TCh, thiocholine; DTNB, 5,5′-dithiobis-(2-nitrobenzoic acid); TNB, 5-dithiobis nitrobenzoic acid; ST, sulfathiazole.

In this study, a colorimetric method was established for Cu^2+^, ST, and glucose detection based on the AChE-like activity of ZIF-8, which may be due to the similarities in structure of them. ZIF-8 consists of a central zinc ion (Zn^2+^) and four 2-methylimidazole ligands. And the crystal structure of AChE indicates that the active site, constituted by a catalytic triad and a so-called anionic subsite, is located at the bottom of a deep and narrow gorge. The anionic subsite recognizes the quaternary ammonium group of the substrate, while the catalytic triad, formed by Glu327, His440, and Ser200, is responsible for the hydrolysis of substrate acetylthiocholine chloride (ATCh) [15] (Figure S1). It is worth mentioning that the process utilizes the enzyme-like activity of the material will not change its structure before and after the reaction. As shown in Figure 1, ZIF-8 can act on the ATCh to generate thiocholine (TCh), which can further react with 5,5′-dithiobis (2-nitrobenzoic acid) (DTNB) to generate 2-nitro-5-thiobenzoic acid (TNB), showing a maximum absorption peak at 405 nm. The reaction can be suppressed by the addition of Cu^2+^, which can chelate with TCh to reduce its reaction to DTNB. Furthermore, gluconic acid and H_2_O_2_ are generated by the reaction of glucose oxidase (GOX) and glucose. The H_2_O_2_ can oxidize TCh, therefore, less TCh can react with DTNB to generate TNB. In addition, ST and its isosteres (sulfamides and sulfamates) have been extensively studied as potent metabolic AChE inhibitors, which will weaken the catalytic reaction of the enzyme [16]. Therefore, a colorimetric method based on the AChE-like activity of ZIF-8 can be designed to detect Cu^2+^, ST, and glucose. The linear relationship between the Cu^2+^, ST, and glucose concentrations and the inhibition rate of ZIF-8 activity was investigated. Finally, the developed method was applied to detect Cu^2+^, ST, and glucose in lake water, milk, and strawberry samples, respectively.

## 2. Results and Discussion

### 2.1. Characterization of ZIF-8 and Feasibility of the Method for Cu^2+^, ST, and Glucose Detection

**Figure 2:**
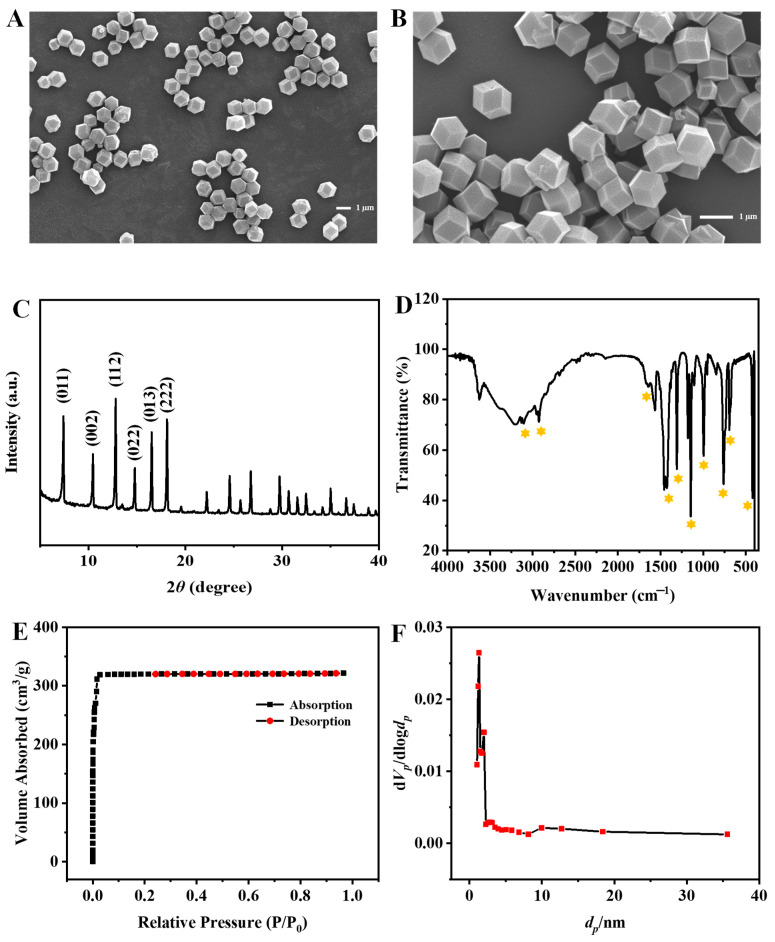
SEM images (**A**,**B**), XRD spectrum (**C**), FT-IR spectrum (**D**) of ZIF-8, nitrogen adsorption-desorption isotherm (**E**) and pore-size distribution (**F**).

The morphology and structure of ZIF-8 were characterized by scanning electron microscope (SEM), X-ray powder diffraction (XRD), Fourier transform infrared spectroscopy (FT-TR), and Brunner-Emmet-Teller (BET) measurements. As the SEM images shown in Figure 2A,B, the synthesized ZIF-8 nanocrystals are rhombic dodecahedrons with a uniform size of about 1 µm. The XRD spectrum depicted in Figure 2C shows mainly sharp and intense diffraction peaks (2*θ* = 011°, 002°, 112°, 022°, and 222°), revealing the formation of a crystalline nanostructure, which is consistent with the previous report [17]. In addition, as shown in Figure 2D, the FT-IR spectrum displays the characteristic bands of ZIF-8. The absorption peaks at 3105 cm^−1^ and 2926 cm^−1^ are due to the NH and CH stretching vibrations, respectively. The absorption peak at 1574 cm^−1^ is assigned to the CN stretching vibration. The absorption peaks at 1425 cm^−1^ and 1308 cm^−1^ correspond to the bending signals of the imidazole ring, and the band at 401 cm^−1^ is attributed to the Zn-N stretching vibration [18]. The ZIF-8 exhibits a BET specific surface area of 1455.6 m^2^/g with a total pore volume of 0.4975 cm^3^/g and an average pore diameter of 1.3671 nm (Figure 2E,F). The above results indicate that ZIF-8 is successfully synthesized.

**Figure 3:**
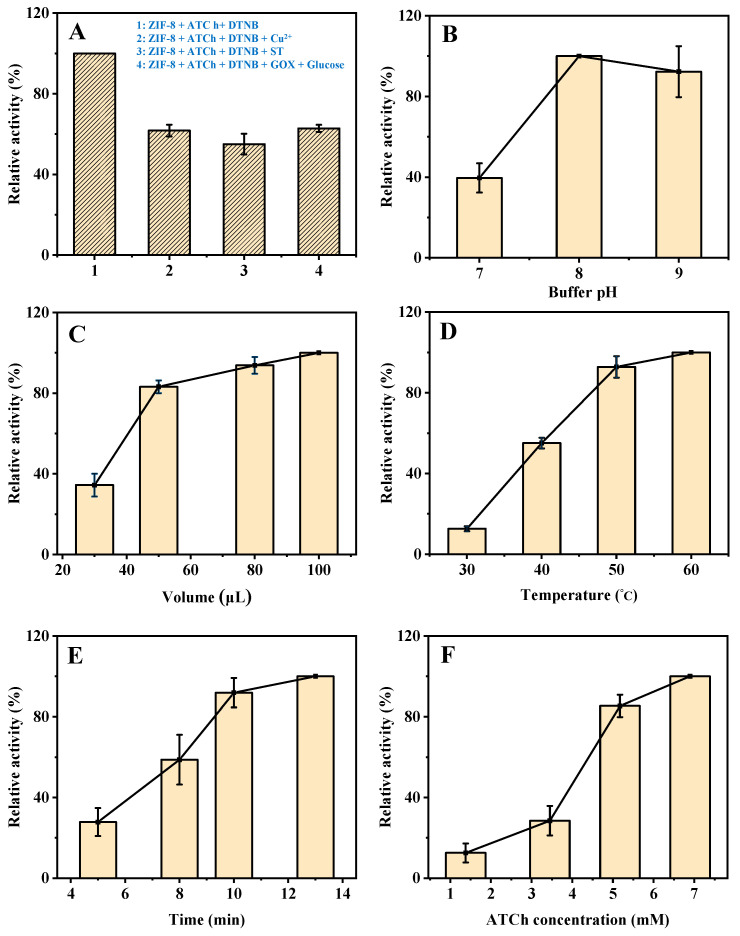
The UV absorption (405 nm) of (**A**-**1**) ZIF-8 + ATCh + DTNB, (**A**-**2**) ZIF-8 + ATCh + DTNB + Cu^2+^, (**A**-**3**) ZIF-8 + ATCh + DTNB + ST, (**A**-**4**) ZIF-8 + ATCh + DTNB + GOX + glucose. Effects of buffer pH (**B**), the volume of ZIF-8 (**C**), reaction temperature (**D**), reaction time (**E**), and the concentration of ATCh (**F**) on the AChE-like activity of ZIF-8. Buffer pH, 8.0 for (**A**,**C**–**F**); the volume of ZIF-8, 30 µL for B, 80 µL for (**A**,**D**–**F**); reaction temperature, 40 °C for (**B**,**C**), 50 °C for (**A**,**E**,**F**); reaction time, 8 min for (**B**–**D**), 10 min for (**A**,**F**); ATCh concentration, 1.51 mM for (**B**–**E**), 5.17 mM for (**A**); Other conditions: DTNB concentration, 1.38 mM; centrifugation time, 2 min; GOX initial concentration, 2 mg/mL; Cu^2+^, 21.55 µM; ST, 344.83 µM; glucose, 0.17 mM.

As shown in Figure S2, the mixture of 2-methylimidazole, ATCh, and DTNB shows a certain absorption at 405 nm. However, during the synthesis process of ZIF-8, the material will be washed twice with deionized water to remove the unreacted 2-methylimidazole, so its impact on the reaction can be ignored. In addition, the mixture of C_4_H_6_O_4_Zn∙2H_2_O, ATCh, and DTNB shows a negligible effect on the reaction as compared to the mixture of ZIF-8, ATCh, and DTNB. As shown in Figure 3A, the mixture of ZIF-8, ATCh, and DTNB shows an obvious absorption at 405 nm. When the Cu^2+^, ST, or GOX and glucose being added to the system, the relative activity of ZIF-8 is obviously weakened. The Cu^2+^ slows down the reaction due to its competitive binding with the intermediate TCh [19]. Furthermore, ST weakens the catalytic reaction of ZIF-8 by affecting its enzyme-like activity. In addition, GOX reacts with glucose to generate highly oxidizing H_2_O_2_, which can act with the intermediate TCh to reduce its reaction to DTNB. Therefore, these results indicate that this AChE-like activity is coming from ZIF-8, and the established method based on the AChE-like activity of ZIF-8 used for the Cu^2+^, ST, and glucose detection is practicable.

### 2.2. Optimization of the Detection Conditions

In order to obtain a good sensitivity of the developed method for Cu^2+^, ST, and glucose detection, several experimental parameters were optimized, including buffer pH, the volume of ZIF-8, reaction temperature, reaction time, and the concentration of ATCh. As shown in Figure 3B, the catalytic activity of ZIF-8 changes with the buffer pH (from 7 to 9), and reaches the highest at pH 8, which was selected for the subsequent experiments (The maximum point in each curve was set as 100%).

Then, the effect of the volume of ZIF-8 was investigated. The catalytic activity increases with the increase in the volume of ZIF-8 from 30 to 100 µL (Figure 3C) but increases slowly from 50 µL and remains stable after 80 µL. Therefore, 80 µL was selected for the subsequent experiments. Furthermore, as shown in Figure 3D, the effect of reaction temperature on the catalytic activity of ZIF-8 was investigated. As the increase in reaction temperature from 30 to 60 °C, the catalytic activity keeps steady above 50 °C, which was selected as the optimum reaction temperature. In addition, the catalytic activity increases with the increase in reaction time (5, 8, 10, and 13 min), and increases slowly after 10 min (Figure 3E). Therefore, further experiments were performed for 10 min. Finally, the effect of the concentration of ATCh was investigated. The catalytic activity increases with the increase in ATCh concentration (1.40, 3.45, 5.17, and 6.90 mM), and increases slowly after 5.17 mM (Figure 3F). Therefore, 5.17 mM of substrate (ATCh) is enough for the reaction. In summary, the optimized reaction conditions for the detection of Cu^2+^, ST, and glucose based on the AChE-like activity of ZIF-8 are as follows. The buffer pH, volume of ZIF-8, reaction temperature, reaction time, and the concentration of ATCh are 8, 80 µL, 50 °C, 10 min, and 5.17 mM, respectively.

### 2.3. Kinetics Study and Stability of ZIF-8

**Figure 4:**
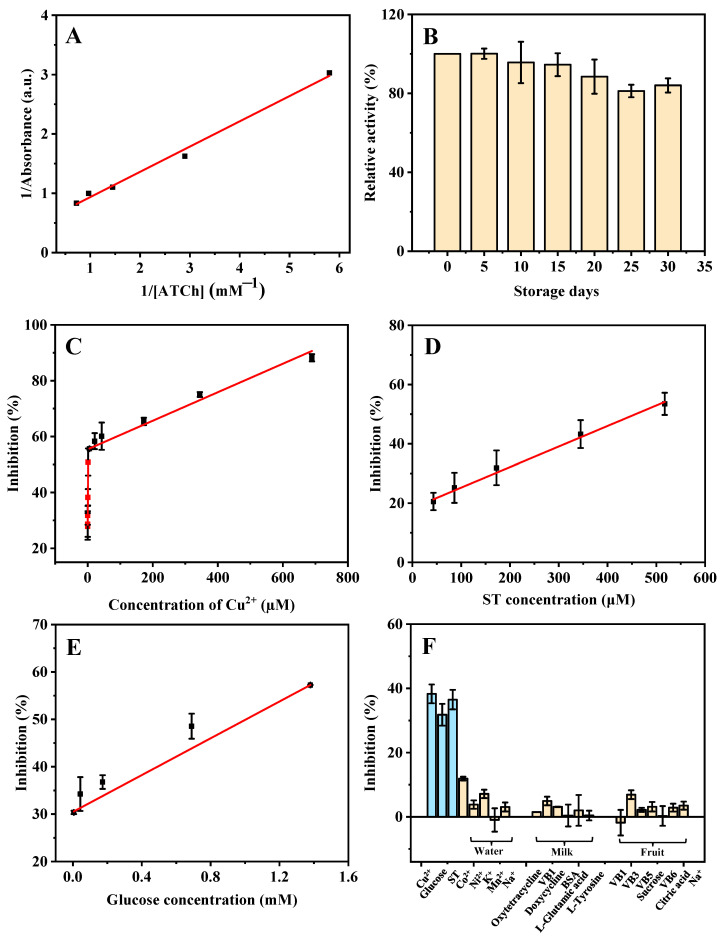
Double reciprocal plot (**A**) and storage stability (**B**) of the ZIF-8 catalyzed activity. The linear range of Cu^2+^ (**C**), ST (**D**), and glucose (**E**), and the selectivity (**F**) for the detection of Cu^2+^, ST, and glucose based on the AChE-like activity of ZIF-8 under the optimized conditions. ATCh concentrations are from 0.17 to 1.40 mM for (**A**). Cu^2+^ concentrations are from 0.021 to 689.66 µM for (**C**). ST concentrations are from 43.10 to 517.24 µM for (**D**). Glucose concentrations are from 0.0054 to 1.40 mM for (**E**). Cu^2+^, ST, and glucose concentrations are 0.67 µM, 172.41 µM, and 0.043 mM, respectively; other possible ions in lake water (Co^2+^, Ni^2+^, K^+^, Mn^2+^, Na^+^) are 344.82 µM; other possible substances in milk (oxytetracycline, doxycycline, VB1, L-glutamic acid, BSA, and L-tyrosine) are 172.41 µM; other possible substances in strawberry (VB1, VB3, VB5, VB6, sucrose, citric acid, and Na^+^) are 344.83 µM for (**F**). Other conditions: buffer pH, 8; the volume of ZIF-8, 80 µL; reaction temperature, 50 °C; reaction time, 10 min; ATCh concentration, 5.17 mM; DTNB concentration, 1.38 mM.

The steady-state kinetic study for the AChE-like activity of ZIF-8 was performed. As shown in Figure 4A, a typical Lineweaver-Burk plot curve was obtained based on the different ATCh concentrations (0.17, 0.34, 0.69, 1.03, and 1.38 mM). Through Equation (1), the obtained linear regression equation of the Lineweaver-Burk plot is y = 0.4256x + 0.5099 and R^2^ = 0.9927, where x and y are the reciprocal of ATCh (substrate) concentration and reaction velocity (the absorbance of product), respectively. A smaller *K_m_* value indicates a stronger affinity between the enzyme and the substrate. The *K_m_* value of the AChE-like activity of ZIF-8 in this study (0.83 mM) is similar to that of natural AChE (0.23 mM) [20], which indicates that the ZIF-8 has a good affinity toward ATCh and exhibits excellent AChE-like catalytic activity. In addition, a batch of ZIF-8 was synthesized and kept at room temperature for 30 days under dry conditions. The relative AChE-like activity of ZIF-8 maintains more than 80.0% of its initial activity after 30 days of storage, and the relative standard deviation (RSD) of batch-to-batch (*n* = 3) is 5.1%, indicating that the synthesized ZIF-8 is stable (Figure 4B).

### 2.4. Colorimetric Detection of Cu^2+^, ST, and Glucose

In Figure 4C, based on the AChE-like activity of ZIF-8, with the increase in concentration of Cu^2+^, the absorbance at 405 nm is decreased because the inhibition rate of ZIF-8 activity is increased. Good linear dependence is obtained in the ranges of 0.021–1.34 µM (calibration curve: y = 16.3204x + 28.0815, R^2^ = 0.9884), and 5.38–689.66 µM (y = 0.05109x + 55.4258, R^2^ = 0.9904). The LOD is calculated based on 3*σ*/*s*, where *σ* is the standard deviation of the blank signal (*n* = 11) and *s* is the slope of the regression line. The LOD is calculated to be 20.00 nM. As shown in Figure 4D, good linear dependence is obtained based on the AChE-like activity of ZIF-8 for the ST detection in the range of 43.10–517.24 µM (calibration curve: y = 0.06929x + 18.2985, R^2^ = 0.9953), and the LOD is calculated to be 9.25 µM. In addition, a linear equation for the detection of glucose is obtained in the range of 0.0054–1.40 mM (calibration curve: y = 19.4178x + 30.4700, R^2^ = 0.9987). The LOD is calculated to be 5.24 µM (Figure 4E). As compared with previous studies (Table 1, Table 2 and Table 3), most of the reported methods require a long time for the synthesis of materials, or a high temperature, and many organic reagents, which is not cost-effective and environmentally friendly. The developed method in this study for the detection of Cu^2+^, ST, and glucose shows some advantages, such as simplicity of the material synthesis process, high efficiency, low LOD, and wide linear range.

For the purpose of assessing the selectivity of the sensing system for Cu^2+^, ST, and glucose detection, some potential interfering substances, including amino acids, small biomolecules, and common ions, were systematically investigated in their effects on the detection. As shown in Figure 4F, the potential substances in the lake water samples (detection of Cu^2+^) such as Co^2+^, Ni^2+^, K^+^, Mn^2+^, and Na^+^, the potential substances in the milk sample (detection of ST) such as oxytetracycline, VB1, doxycycline, BSA, L-glutamic acid, and L-tyrosine, the potential substances in fruit sample (detection of glucose) such as VB1, VB3, VB5, VB6, sucrose, citric acid, and Na^+^, were investigated. The results indicate that only Cu^2+^, ST, and glucose have obvious effects on the AChE-like activity of ZIF-8, indicating that the sensor has a high specificity for the detection of Cu^2+^, ST, and glucose.

### 2.5. Detection of Cu^2+^, ST, and Glucose in Real Samples

To evaluate the practical application of this sensing platform, the real samples were analyzed on the basis of the standard addition method. As shown in Table 4, the established method was used for the detection of Cu^2+^, ST, and glucose in the lake water, milk, and strawberry samples. A standard addition method was adopted by adding Cu^2+^ into the lake water samples to reach the final concentrations of 0.084, 21.55, and 172.41 µM, and the sample spiked recoveries are within the range of 81.4–112.8% with the RSD (*n* = 3) lower than 8.1%. Furthermore, the ST was added into the milk sample with the final concentrations of 43.10, 86.21, and 172.41 µM, and analyzed by the developed method, the sample spiked recoveries are in the range of 98.4–115.4% with the RSD lower than 3.3%. Finally, standard addition of glucose to the strawberry sample with the final concentration of 0.086, 0.17, and 0.34 mM was performed, the sample spiked recoveries are in the range of 93.0–112.8% with the RSD lower than 1.8%. These results indicate that the developed method has good analytical performance and can be applied in the real sample analysis. In addition, this Cu^2+^, ST, and glucose sensor shows reasonable repeatability and reliability for their low RSD% value.

## 3. Materials and Methods

### 3.1. Chemicals and Materials

Potassium chloride (KCl), sodium phosphate dibasic dihydrate (NaH_2_PO_4_∙2H_2_O), and sodium chloride (NaCl) were purchased from Chengdu Chron Chemicals Co., Ltd. (Chengdu, China). Copper (II) sulfate pentahydrate (CuSO_4_·5H_2_O) was purchased from Chongqing Chuandong Chemical Group Co., Ltd. (Chongqing, China). D (+)-glucose, 2-methylimidazole, vitamin B1 (VB1), vitamin B6 (VB6), vitamin B3 (VB3), vitamin B5 (VB5), ST, and ATCh (≥99%) were purchased from Shanghai YuanYe Biological Technology Co., Ltd. (Shanghai, China). DTNB, GOX, and zinc acetate dihydrate (C_4_H_6_O_4_Zn∙2H_2_O) were purchased from Macklin (Shanghai, China). L (+)-Glutamic acid and manganese chloride (MnCl_2_∙4H_2_O) were purchased from Shanghai Aladdin Biochemical Technology Co., Ltd. (Shanghai, China). L-tyrosine was purchased from Chengdu Huaxia Chemical Reagent Co., Ltd. (Chengdu, China). Bovine serum albumin (BSA) was purchased from Sangon Biotech Co., Ltd. (Shanghai, China). All the chemicals are analytical grade and deposited at the Pharmaceutical Engineering Laboratory of School of Chemistry and Chemical Engineering, Chongqing University (Chongqing, China). The milk sample was purchased from Chongqing Tianyou Dairy Co., Ltd. (Chongqing, China). The real water samples were obtained from the YUN and JIN lakes of Chongqing University (Chongqing, China). Strawberry was purchased from Chongqing New Century Department Store (Chongqing, China).

### 3.2. Instruments

For the characterization of material, the SEM images were obtained through a field-emission SEM (JSM-7600F, JEOL Ltd., Tokyo, Japan); The XRD pattern was obtained using a X’ pert Powder diffractometer (Malvern Panalytical Ltd., Malvern, The Netherlands) with secondary beam graphite monochromated Cu Kα radiation; The FT-TR pattern was obtained through a Nicolet iS50 (Thermo Scientific Inc., Waltham, MA, USA); And the BET studies were performed on a Quadrasorb 2 MP (Kantar, New York, NY, USA) specific surface and aperture analyzer. The ultrapure water used for all experiments was purified by a water purification system (ATSelem 1820A, Antesheng Environmental Protection Equipment, Chongqing, China). The UV-Vis analysis was performed on a UV-5500 PC spectrophotometer (Shanghai Metash Instruments Co., Ltd., Shanghai, China). A tabletop low-speed centrifuge L420 was purchased from Hunan Xiang Yi Laboratory Instrument Development Co., Ltd. (Changsha, China).

### 3.3. Synthesis of ZIF-8

The ZIF-8 was synthesized according to the previously reported method [41]. Firstly, the mixture solution of 4 mL of C_4_H_6_O_4_Zn∙2H_2_O (0.40 M) and 40 mL of 2-methylimidazole (0.80 M) was placed in an oven and reacted at 30 °C for 2 h. The obtained ZIF-8 was purified by centrifugation for 5 min at 3800 rpm (2259× *g*) and rinsed with ultra-pure water twice. Finally, the prepared ZIF-8 was re-suspended in 8 mL of deionized water and stored in a 4 °C refrigerator.

### 3.4. ZIF-8 Activity Assays

ZIF-8 can catalyze ATCh to TCh, which will further react with DTNB to generate TNB that has a maximum absorption peak at 405 nm. In a 0.5 mL centrifuge tube, 80 μL of ZIF-8 (dispersed in deionized water), 100 μL of PBS (prepared in 10.00 mM of phosphate buffer, pH 8), 200 μL of 5.17 mM ATCh (prepared in 10.00 mM phosphate buffer, pH 8), and 200 μL of 1.38 mM DTNB (prepared in ethanol) were mixed together, and then incubated at 50 °C with shaken at 160 rpm for 10 min. After being centrifuged by a handheld mini centrifuge for 2 min, the absorbance of the supernatant at 405 nm was recorded. Each sample was measured three times.

### 3.5. Enzyme Kinetics

The *K_m_* is an important parameter of an enzyme kinetic reaction and reflects the affinity between the enzyme and substrate, which can be calculated by the Lineweaver-Burk Equation (1) [42].

(1) $$\frac{1}{V}=\frac{K_{m}}{V_{max}\left[S\right]}+\frac{1}{V_{max}}$$

where *V* and *V_max_* are the initial and maximum rate of the enzyme reaction, respectively, [*S*] is the concentration of the substrate (ATCh), and *K_m_* is the Michaelis-Menten constant. The initial velocity of ZIF-8 was monitored by the absorbance of the product (TNB). Experiments were carried out by altering the concentration of ATCh (0.17, 0.34, 0.69, 1.03, and 1.40 mM) under optimized conditions.

### 3.6. Procedure for Cu^2+^, ST, and Glucose Determination

Based on the AChE-like activity of ZIF-8, a facile colorimetric method for Cu^2+^, ST, and glucose detection was developed. For the detection of Cu^2+^, 80 μL of ZIF-8, 100 μL of different concentrations of Cu^2+^ (0.021, 0.042, 0.17, 0.67, 1.35, 5.39, 21.55, 43.10, 172.41, 344.83, and 689.66 µM), 200 μL of 5.17 mM ATCh (prepared in 10.00 mM phosphate buffer, pH 8), and 200 μL of 1.38 mM DTNB (prepared in ethanol) were mixed together into a 0.5 mL centrifuge tube, and then incubated at 50 °C with shaken at 160 rpm for 10 min, respectively. For the detection of ST, 80 μL of ZIF-8, 100 μL of different concentrations of ST (43.10, 86.21, 172.41, 344.83, and 517.24 µM), 200 μL of 5.17 mM ATCh (prepared in 10.00 mM phosphate buffer, pH 8), and 200 μL of 1.38 mM DTNB (prepared in ethanol) were mixed together into a 0.5 mL centrifuge tube, and then incubated at 50 °C with shaken at 160 rpm for 10 min, respectively. For the detection of glucose, 80 μL of ZIF-8, 50 μL of different concentrations of glucose (0.0054, 0.043, 0.17, 0.69, and 1.40 mM), 50 μL of GOX (initial concentration of 2 mg/mL), 200 μL of 5.17 mM ATCh (prepared in 10.00 mM phosphate buffer, pH 8), and 200 μL of 1.38 mM DTNB (prepared in ethanol) were mixed together into a 0.5 mL centrifuge tube, and then incubated at 50 °C with shaken at 160 rpm for 10 min, respectively. The absorbance at 405 nm of the mixture supernatant was recorded. Each sample was measured three times.

### 3.7. Real Sample Analysis

To validate its feasibility and practicability, the method based on the AChE-like activity of ZIF-8 was used to detect the Cu^2+^, ST, and glucose in lake water, milk, and strawberry samples, respectively. (1) For the Cu^2+^ detection, 80 μL of ZIF-8, 100 μL of different concentrations of Cu^2+^ (final concentrations of 0.084, 21.55, and 172.41 μM) spiked lake water, 200 μL of 5.17 mM ATCh (prepared in 10.00 mM phosphate buffer, pH 8), and 200 μL of 1.38 mM DTNB (prepared in ethanol) were mixed together into a 0.5 mL centrifuge tube, and then incubated at 50 °C with shaken at 160 rpm for 10 min, respectively. (2) For the ST detection, 80 μL of ZIF-8, 100 μL of different concentrations of ST (final concentrations of 43.10, 86.21, and 172.41 μM) spiked milk sample, 200 μL of 5.17 mM ATCh (prepared in 10.00 mM phosphate buffer, pH 8), and 200 μL of 1.38 mM DTNB (prepared in ethanol) were mixed together into a 0.5 mL centrifuge tube, and then incubated at 50 °C with shaken at 160 rpm for 10 min, respectively. (3) For the glucose detection, in a 0.5 mL centrifuge tube, 80 μL of ZIF-8, 50 μL of GOX (initial concentration of 2 mg/mL), 50 μL of different concentrations of glucose (final concentrations of 0.086, 0.17, and 0.34 mM) spiked strawberry sample, 200 μL of 5.17 mM ATCh (prepared in 10.00 mM phosphate buffer, pH 8), and 200 μL of 1.38 mM DTNB (prepared in ethanol) were mixed well, and then incubated at 50 °C with shaken at 160 rpm for 10 min, respectively. The absorbance at 405 nm of the mixture supernatant was recorded. The spiked recoveries in lake water, milk, and strawberry samples of Cu^2+^, ST, and glucose were calculated by the linear relationship between the inhibition rates and their concentrations, respectively.

## 4. Conclusions

In summary, the AChE-like activity of ZIF-8 was successfully applied in the sensitive detection of Cu^2+^, ST, and glucose. ZIF-8 has high AChE activity and shows good affinity to the substrate, which also has good storage stability. As compared with the other methods, this method has the advantages of a low LOD and wide detection range, which has the potential as a convenient method for the rapid and sensitive detection of Cu^2+^, ST, and glucose. Moreover, the ZIF-8 is of high stability, and low cost, which can be synthesized through a simple method under mild conditions. In addition, ZIF-8 has a series of enzyme-like activities, which can be further applied in the analysis of different analytes. In short, this study provides a feasible detection method for Cu^2+^, ST, and glucose, which may be applied in monitoring their levels in some health problems, such as Alzheimer’s disease, Menke’s disease, Wilson’s disease, resistance to antibiotics, allergic reactions, and toxic effects, and Diabetes.

## Figures and Tables

**Table 1:** Comparison of the previously reported methods and present work for the detection of Cu^2+^.

Material	Detection Method	Material Synthesis Process	Reaction Time (min)	Linear Range (µM)	LOD (µM)	Ref.
Ag/Zn-ZIF-8	Fluorescence	Synthesized at room temperature for 20 h–24 h; dried overnight in a vacuum oven at 80 °C	30	1–20	6.7	[21]
ZnO-Co_3_O_4_	Colorimetry	Synthesized at room temperature for 24 h; dried in vacuum at 60 °C for 12 h; heated at 350 °C for 3 h	10	2–100	1.1	[22]
5-chlorosalicylaldehyde fluorescein hydrazone (CSFH)	Colorimetry	Refluxed for 8 h under constant stirring	0.17	0.25–14	0.3	[23]
1-Phenyl-3-methyl-5-hydroxypyrazole-4-benzoyl (fluorescein) hydrazone	Colorimetry	Settle for 2 h; refluxed for 8 h with stirring	2	2–40	2	[24]
Au NPs	Colorimetry	Heated to boiling under vigorous stirring; heated for an additional 30 min	10	0.1–1.2	0.05	[25]
h-CD	Fluorescence	Heated to 180 °C and kept for 12 h; dialyzed with a dialysis membrane (molecular weight of 7000 Da) for 12 h	720	0–10	0.2	[26]
P(DO-a-DTODT)-g-BTDX	Colorimetry	Stirred continuously for 12 h	-	0–10	0.03	[27]
Pt/Co_3_O_4_	Colorimetry	Synthesized at 160 °C for 1 h; dried in vacuum at 50 °C for 12 h; calcined in a muffle furnace at 450 °C for 4 h; synthesized at 90 °C for 20 min under constant stirring	15	10–200	4.1	[28]
ZIF-8 (AChE)	Colorimetry	Synthesized at 30 °C for 2 h	10	0.021–1.34; 5.38–689.66	0.020	This work

Au NPs: gold nanoparticles; CD: carbon dots; P(DO-a-DTODT): poly (4-dioxa-alt-2,7dithiaoctadecane-4,5,9,16-tetraol); BTDX: 2-(6-bromobenzo[d]thiazol-2-yl)-3′,6′-bis(diethylamino)spiro[isoindoline-1,9′-xanthen]-3-one; AChE: acetylcholinesterase.

**Table 2:** Comparison of the previously reported methods and present work for the detection of ST.

Material	Detection Method	Material Synthesis Process	Reaction Time (min)	Linear Range (U/L)	LOD (U/L)	Ref.
μPAD-microfluidic paper	Colorimetry	Heated for 2 min at a temperature of 150 °C	15	2.5–40	2.5	[29]
AuNPs	Surface-enhanced Raman spectroscopy	Keep boiling and stirring for 30 min	0.17	2.0–78.3	3.9	[30]
Cu NCs	SPME-Fluorescence	Refluxed for 15 h; degassed by nitrogen for 30 min and then heated at 70 °C for 24 h	55	0.5–99.9	0.3	[31]
-	HPLC- Fluorescence	-	100	29.4–1175.0	9.8	[32]
MIP/CuS/Au@COF/GCE	Electrochemistry	Heated for 10 h at 120 °C; stirred for 12 h at room temperature	-	1.0–10^8^	4.3	[33]
MIP/MCL	Chemiluminescence	Stirred for 5 min and kept at 4 °C overnight; stirred in a 65 °C water bath for 24 h; extracted with methanol/acetic acid for successive 72 h	20	3.9–47.0	3.9	[34]
ZIF-8 (AChE)	Colorimetry	Synthesized at 30 °C for 2 h	10	43.10–517.24	9.25	This work

μPAD: paper-based analytical device; AuNPs: gold nanoparticles; Cu NCs: copper nanoclusters; MIP: molecularly imprinted polymers; COF: covalent organic frameworks; GCE: glassy carbon electrode; MCL: microtiter chemiluminescence; AChE: acetylcholinesterase.

**Table 3:** Comparison of the previously reported methods and present work for the detection of glucose.

Material	Detection Method	Material Synthesis Process	Reaction Time (min)	Linear Range (mM)	LOD (µM)	Ref.
AgNC-GOx/Ag^+^-FP	Fluorescence	Synthesized at 150 °C for 20 min	120	0.050–5	50.0	[35]
g-C_3_N_4_	Electrochemistry	Calcined at 550 °C for 2 h; heated at 500 °C for 2 h	-	0.050–2	5.0	[36]
ZnONT	Electrochemistry	Synthesized at 85 °C for 1.9 h; kept at 85 °C for 1.5 h	-	0.050–12	1.0	[37]
GOx@CPB	Colorimetry	Synthesized at 60 °C for 5 min	1	0.0005–0.1	0.5	[38]
Co-MOFs	Electrochemistry	Synthesized at 100 °C for 72 h	-	0.010–1.2	3.2	[39]
ZIF-67/rGO/CF	Electrochemistry	Treated at 300 °C for 30 min; kept at room temperature for 4 h	-	0.001–1	0.2	[40]
ZIF-8 (AChE)	Colorimetry	Synthesized at 30 °C for 2 h	10	0.0054–1.40	5.24	This work

AgNC: silver nanocube; GOx: glucose oxidase; FP: fluorescence probe; g-C_3_N4: graphitic carbon nitride; ZnONT: zinc oxide nanotubes; CPB: copper analogue of Prussian blue; rGO: reduced graphene oxide; CF: carbon fibers; AChE: acetylcholinesterase.

**Table 4:** Determination of Cu^2+^, ST, and glucose in lake water, milk, and fruit samples, respectively.

Substance	Sample	Added (µM)	Founded (µM)	Recovery (%)	RSD (*n* = 3, %)
Cu^2+^	YUN lake	0.084	0.098	116.7	1.5
		21.55	23.22	107.7	3.4
		172.41	192.27	111.5	2.7
	JIN lake	0.084	0.075	89.3	1.5
		21.55	17.68	82.0	8.1
		172.41	183.19	106.3	6.1
ST	Milk (Tianyou)	43.10	42.41	98.4	1.8
		86.20	99.47	115.4	3.3
		172.41	196.89	114.2	1.9
Glucose	Strawberry	0	0.95	-	2.8
		0.086	1.03	93.0	0.9
		0.17	1.12	100.0	1.8
		0.34	1.33	111.8	1.2

## Data Availability

The data presented in this study are contained within the article.

## Supplementary Materials

The following supporting information can be downloaded at: https://www.mdpi.com/article/10.3390/molecules27217491/s1, Figure S1: Structures of AChE and ZIF-8; Figure S2: The UV absorption (405 nm) of (1) 2-methylimidazole + ATCh + DTNB, (2) C_4_H_6_O_4_Zn∙2H_2_O + ATCh + DTNB, (3) ZIF-8 + DTNB, (4) ZIF-8 + ATCh + DTNB. Buffer pH, 8.0; the volume of ZIF-8, 80 µL; reaction temperature, 50 °C; ATCh concentration, 5.17 mM; DTNB concentration, 1.38 mM; centrifugation time, 2 min; 2-methylimidazole concentration, 17.2 mM; C_4_H_6_O_4_Zn∙2H_2_O concentration, 17.2 mM.

## Sample Availability

Samples of the compounds are available from the authors.
