# Supplementary material for: A Sensitive and Selective Colorimetric Method Based on the Acetylcholinesterase-like Activity of Zeolitic Imidazolate Framework-8 and Its Applications
Source: Molecules. 2022 Nov 3;27(21):7491. doi: 10.3390/molecules27217491 (PMC9656881; doi:10.3390/molecules27217491)
Supplement: Supplementary file 1 [file molecules-27-07491-s001.zip › molecules-1975578-supplementary.pdf]

Supplementary Material

A sensitive and selective colorimetric method based on the acetylcholinesterase-like activity of zeolitic imidazolate framework-8 and its applications

Guo-Ying Chen [1], Zheng-Ming Qian [2, 3*], Shi-Jun Yin [1], Xi Zhou [1], Feng-Qing Yang [1*]

[1] School of Chemistry and Chemical Engineering, Chongqing University, Chongqing 401331, China

[2] College of Medical Imagine Laboratory and Rehabilitation, Xiangnan University, Chenzhou 423000, China.

[3] Dongguan HEC Cordyceps R&D Co., Ltd., Guangdong 523850, China.

* Correspondence: Feng-Qing Yang, Phone number: +86-13617650637. E-mail: fengqingyang@cqu.edu.cn. Zheng-Ming Qian, Xiangnan University, Chenzhou 423000, E-mail: qianzhengming1982@126.com.

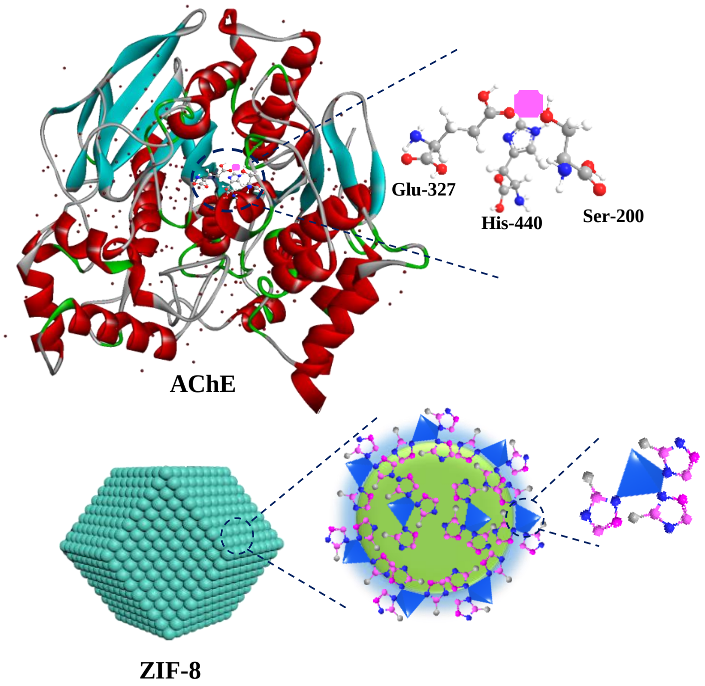

Figure S1. Structures of AChE and ZIF-8.

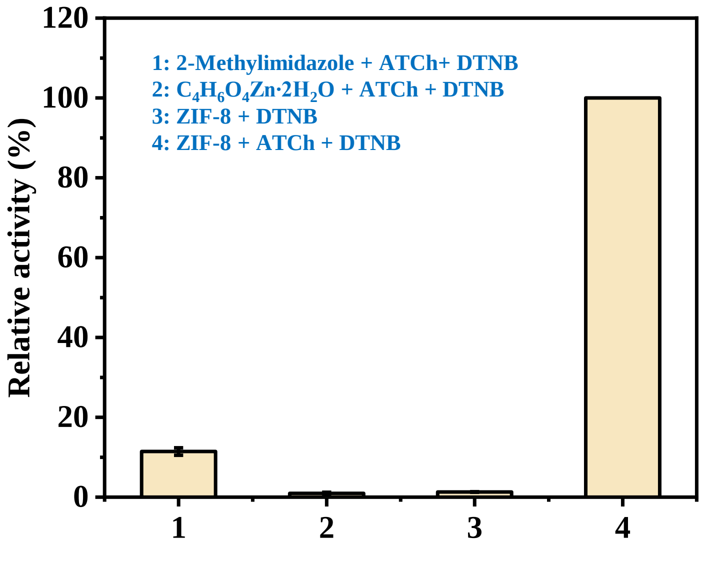

Figure S2. The UV absorption (405 nm) of (1) 2-methylimidazole + ATCh + DTNB, (2) $C_4H_6O_4Zn{\cdot}2H_2O$ + ATCh + DTNB, (3) ZIF-8 + DTNB, (4) ZIF-8 + ATCh + DTNB. Buffer pH, 8.0; the volume of ZIF-8, 80 μL; reaction temperature, 50°C; ATCh concentration, 5.17 mM; DTNB concentration, 1.38 mM; centrifugation time, 2 min; 2-methylimidazole concentration, 17.2 mM; $C_4H_6O_4Zn{\cdot}2H_2O$ concentration, 17.2 mM.
